# Control of organ size: development, regeneration, and the role of theory in biology

**DOI:** 10.1186/s12915-015-0123-7

**Published:** 2015-02-19

**Authors:** Charles F Stevens

**Affiliations:** The Salk Institute, 10010 North Torrey Pines Rd, La Jolla, CA 92037 USA

## Abstract

How organs grow to be the right size for the animal is one of the central mysteries of biology. In a paper in *BMC Biology*, Khammash *et al*. propose a mechanism for escaping from the deficiencies of feedback control of growth as a mechanism.

See research article: http://dx.doi.org/10.1186/s12915-015-0122-8

## Commentary

When I read the new paper by Buzi, Lander, and Khammash [1], two things immediately caught my eye. I was attracted by the science, but I was also interested by issues the paper tacitly raises about the role of theory in biology. First, the science.

One of the central problems in biology is how the size of organs is controlled during development and regeneration. The field of allometry is based on the observation that the sizes of various organs are always appropriate for the animal size, but how this is achieved during growth remains mysterious. The goal of the paper is to explore, using theory, two ways organ size could be controlled.

A theory is often taken to be a possibly insubstantial conjecture (‘Oh, that’s just a theory’), but the basis of the theory used in this paper is no more than the observation that when a cell divides it produces two daughters. Picture the simplest possible way an organ could be produced during growth or regeneration: suppose there are just two cell types, organ cells and the progenitors to make them. Progenitor cells can divide in three different ways: (1) expansive division (two new progenitors per cell division), (2) asymmetric division (one progenitor and one organ cell), or (3) symmetric division (two organ cells). How fast the organ grows depends on what fraction of the progenitors pick each of the three types of cell division. Expansive division would increase the size of the progenitor pool, asymmetric division would maintain the progenitor pool size and increase the organ size, and symmetric division would decrease the organ growth rate by shrinking the progenitor pool size and decreasing the total rate of cell division.

Such a simple scheme would, however, have no control of organ size. Something else is needed. Experimental data, discussed in the paper, support the idea that the something else is a signal organ cells send to the progenitors. This signal causes more progenitors to use the symmetric division mode. If each cell in the growing organ sends such a signal, the strength of the signal will increase with organ size, and this will slow the organ growth by using up progenitors in symmetric divisions. Ultimately, the rate at which the cells in the organ happen to die will be just matched by the rate at which progenitors produce new organ cells, and the organ size will remain fixed. And if part of the organ is removed, decreasing the number of cells sending the signal will cause some progenitors to pick expansive division, the progenitor population will grow, and the growth process just described will repeat to make the organ grow back to its correct size.

This very simple scheme is called by the authors ‘renewal control’ because the growth-control signal from the organ cells regulates what fraction of the progenitors use which cell division mode. It explains how organs can reach a final size, maintain that size, and regenerate back to the same size when part of the organ is removed.

The only problem is that the beautifully simple scheme won’t actually always work. Here is why. The renewal-control signal changes the progenitor cell division mode rapidly, but the corresponding change in size of the organ, and thus in the magnitude of the growth-control signal, takes longer to occur. This means that things like rapid changes in the death rate of organ cells, or random fluctuations in how many progenitors are dividing at any moment can be too fast to be corrected properly by the renewal control mechanisms, the organ growth will be too fast or too slow, and the organ size can continue to vary rather than becoming fixed at the right size. In many cases the simple scheme will work well to produce organs of the correct size, but evolution has to plan for mechanisms for growth control that will work under all circumstances. The renewal control will fail in certain cases and so the simple scheme is an unsuitable evolutionary choice.

The authors have identified a slightly more complicated but still quite simple scheme that keeps the renewal control idea but extends it slightly and fixes the instabilities inherent in renewal control. They call this modified scheme ‘fate control’.

The trick is to suppose that the renewal-control signal also controls a fate choice of the progenitor. In pure renewal control, the progenitors can make only progenitors or organ cells, but in the modified scheme, the progenitors can make three cell types: progenitors, organ cells, and alternative cells. The nature of the alternative cells is unspecified, and what they are actually good for is mostly irrelevant for the argument. Now, the growth control signal from organ cells is also supposed to control the fate chosen by progenitors. By changing the fraction of progenitors that make organ cells verses alternative cells, the link between the rate of change in the size of the progenitor pool and the organ growth rate present in pure renewal control is broken, and the instabilities noted above are eliminated once that link is no longer present.

The reader will have noticed that I used no equations to describe the two types of growth control and the limitations of renewal control. Some theories do not need to, or cannot, be formalized to be useful (think biology’s most important theory, Evolution) but, of course, certain types of theoretical research cannot progress without formalization. Thinking about how a system works is always necessary, but for some theories, quantitative features of the theory must be explored so that the magnitude of effects can be evaluated, and the exact conditions under which they occur can be found. This means that what I have put into words above has been formalized as equations that can be manipulated to get the sorts of answers I have tried to provide above with qualitative arguments. This formalization of the size control system, so it can be quantitatively explored, is exactly what the authors did.

Now for the second thing that caught my attention. One of neurobiology’s most successful theories is embodied in the Hodgkin-Huxley equations that explain how the nerve impulse works [2]. Hodgkin and Huxley described the behavior of ion channels (as we would now say) as a function of membrane voltage and time in experiments that held voltage constant after a step change. Then they described this channel behavior measured in their experiments with equations, and solved the equations with voltage permitted to vary with time rather than remain constant after a voltage step. They knew their theory was correct when their equations correctly predicted how voltage varies during a nerve impulse with values of all parameters determined by experiment. This landmark work established the gold standard, rarely reached, for good mathematical models in biology: the best models accurately predict experimental results with no free parameters.

The present paper, however, does not compare predictions of the theory for controlling organ size with experimental data. Indeed, it does not even make specific predictions that can be compared with data (although it could be reformulated to do so in certain cases). Does this no-prediction approach represent a useful direction for theory in biology?

I would argue that it is a useful direction. Here is why. Sometimes - rarely - you can do experiments that tell you everything you need to make a theory. Hodgkin and Huxley’s experiments and theory are an example of this. But often there is a problem that is very complicated, one that you know enough about to constrain a theory but not enough to define a question you can answer. I believe control of organ size falls in that category. We know enough about the cell cycle, cell division, and developmental biology to constrain answers to the question of how organ size is established, but not enough to formulate a specific set of experiments that will give you an answer. In this case, the field has to define more precisely the question, to develop intuitions about what sort of thing might work, and to make use of the constraints we have to explore possibilities. This paper does just that.
